# Toward a Compositional Theory of Trust in Embodied Intelligence: A QNLP Framework for Modeling Context, Interaction, and Trustworthiness

**DOI:** 10.3390/biomimetics11060438

**Published:** 2026-06-19

**Authors:** Yang Li, Yao Song

**Affiliations:** 1 Youth League Committee, Sichuan University, Chengdu 610065, China; li-yang@scu.edu.cn; 2 Digital Convergence Laboratory of Chinese Cultural Inheritance and Global Communication, Sichuan University, Chengdu 610065, China; 3 Academy of Chinese, History, Religion and Philosophy, Hong Kong Baptist University, Hong Kong SAR, China

**Keywords:** embodied intelligence, human–robot trust, trust calibration, predictive reliability, quantum natural language processing, compositional semantics, clustering analysis, human–AI interaction

## Abstract

Trust in embodied intelligence is dynamic, contextual, and interaction-dependent, but many existing computational approaches still model trust using static similarity structures. This study proposes and evaluates a compositional trust modeling approach based on quantum natural language processing (QNLP). Using open-ended survey responses about human trust in embodied agents, we compared classical NLP clustering and QNLP-based clustering in terms of dimension coverage, semantic coherence, contextual sensitivity, and robustness. The QNLP pipeline captured richer latent structure, producing ten clusters and identifying eight trust dimensions, including two emergent dimensions: calibrated trust and predictive reliability. Compared with classical approaches, QNLP clusters showed improved semantic separation and stronger context retention under preprocessing variation. These findings support a temporally structured view of trust in embodied AI and demonstrate that compositional quantum-inspired representations can reveal nuanced trust dynamics that are difficult to detect with conventional methods. This study contributes both a methodological framework for trust-sensitive text modeling and a theoretical account linking trust formation to retrospective calibration and prospective expectation in human–agent interaction.

## 1. Introduction

In the realm of embodied intelligence, trust plays a pivotal role in facilitating meaningful and effective interactions between humans and intelligent agents [1,2,3]. As embodied AI systems—ranging from autonomous robots to virtual assistants—become increasingly integrated into everyday life, the need for a robust and dynamic framework to model trust is paramount [1]. Trust is essential not only in ensuring that users feel comfortable relying on these systems but also in supporting their ability to predict and respond to a wide array of tasks in real-world contexts [3]. The evolving nature of embodied intelligence, marked by autonomy, unpredictability, and multimodal interactions, necessitates a rethinking of how trust can be represented, calibrated, and measured [4,5].

Despite the critical importance of trust, much of the current literature on trust modeling in intelligent systems has relied heavily on similarity-based approaches, particularly clustering techniques [3,4]. These methods, while offering valuable insights into static relationships, fall short in addressing the complexities of trust in embodied intelligence. Trust judgments are inherently dynamic, shaped by context, interaction history, and the evolving performance of the agent [5,6,7]. Clustering-based models, by their nature, focus on grouping data based on static similarities and are ill-suited to capture the nuances of context-dependent trust, which requires a more compositional and flexible approach [8,9]. Furthermore, these models typically lack the capacity to integrate the multimodal and action-oriented nature of trust in embodied agents, leaving critical dimensions of trust unaccounted for [4,10,11].

This paper makes three key contributions to the field [3,4]. Methodologically, we argue that existing similarity-based models, such as clustering, cannot adequately represent trust in embodied intelligence due to their inability to account for compositionality, context dependence, and evolving trust judgments [5,12]. We propose a more principled approach using QNLP (quantum natural language processing), which allows for the representation of trust in a dynamic, context-sensitive, and interaction-dependent manner [9,13]. Theoretically, this study reconceptualizes trust in embodied intelligence by moving beyond the traditional social-robot paradigm. Rather than being based solely on perceived similarity or simple behavior models, trust in these systems must be understood as an ongoing process of calibration, grounded in transparency, predictive reliability, and context-specific actions [14]. Finally, we present a new framework for trust modeling in embodied intelligence that integrates linguistic signals, action outcomes, multimodal context, and dimensions of trustworthiness into a unified compositional representation. This framework aims to provide a more comprehensive and flexible model for understanding trust in embodied agents, advancing both theoretical perspectives and practical applications in the field [15].

## 2. Literature Review

### 2.1. Trust in Social Robotics and Embodied Intelligence

Trust in social robotics has traditionally been conceptualized as a measure of an agent’s reliability and predictability in performing tasks or following human instructions [4,16]. Early models often emphasized static cues, such as physical appearance, anthropomorphic features, and observable behavioral patterns, assuming that these factors alone could determine a human user’s trust in the robot [17,18]. Metrics like the Facial Anthropomorphic Trustworthiness towards Social Robots (FATSR-17) scale illustrate this approach, quantifying trust based on facial features and perceived human-likeness rather than on actual interactive experience [19,20]. While these measures provided useful insights for early social-robot design, they fail to capture the complexity of trust as it emerges in dynamic, real-world interactions [4,16,17,18,21,22].

With the advent of embodied intelligence systems—autonomous agents capable of perceiving, reasoning, and acting in shared human environments—the nature of trust has shifted dramatically [11,12]. Physical embodiment introduces contextual and relational dimensions to trust [12,22]. A robot’s physical presence in a human workspace or domestic environment creates potential risks, both physical and social, that make human trust contingent not only on task performance but also on the agent’s adherence to social norms, responsiveness to nonverbal cues, and alignment with human goals [12,22]. For example, a robot that fails to interpret a subtle gesture or gaze may inadvertently undermine trust, even if it performs its task correctly [21,23]. Unlike virtual agents, which operate behind a screen and whose failures are largely symbolic, embodied agents interact in shared space, where trust is inseparable from the agent’s ability to act safely, transparently, and predictably in real time [23,24,25,26].

Moreover, human trust in embodied agents is inherently dynamic and context-dependent [7,27]. It evolves across interactions, shaped by the agent’s ability to integrate verbal, nonverbal, and environmental signals into behavior that aligns with human expectations [7,27]. Traditional approaches, which rely on fixed assessments or task-based performance metrics, fail to account for this evolving nature [4,27]. Humans do not form trust based solely on isolated interactions; rather, trust emerges through a continuous feedback loop in which the agent interprets human signals, adjusts its actions, and demonstrates consistent reliability over time [7,27,28,29,30].

Quantum natural language processing (QNLP) offers a methodological approach to address these challenges [13,31]. By modeling multimodal human inputs—such as language, gestures, tone, and contextual cues—as entangled states rather than independent signals, QNLP enables embodied agents to capture the compositional and interaction-dependent structure of trust. This allows for the accurate parsing of complex human intentions and the generation of behavior that maintains behavioral believability, which is central to sustaining trust in embodied systems [9,13]. In this sense, QNLP does not define trust itself but acts as an interpretive engine, supporting the real-time integration of multimodal inputs into actionable behaviors that humans perceive as trustworthy [9,13,31].

In summary, trust in social robotics has moved from a static, anthropomorphism-focused measure to a dynamic, interaction-sensitive construct that depends on compositional, multimodal interpretation [4,22]. Embodied intelligence, with its physical presence and autonomous capabilities, demands computational approaches capable of modeling trust as evolving, contextually grounded, and behaviorally demonstrable—criteria that QNLP is uniquely suited to address [31,32,33].

### 2.2. Existing Approaches to Understanding Trust in Embodied Intelligence

Understanding trust in embodied intelligence requires first clarifying what trust represents in the context of human–agent interaction [3,4]. In classical robotics and early AI systems, trust was often defined narrowly as the expectation that a system would perform a task reliably or correctly [2,3]. Computationally, this was operationalized through measures such as task success rates, adherence to instructions, or consistency of observable behavior. Clustering algorithms, statistical correlation models, and simple probabilistic frameworks were commonly employed to model trust, typically treating it as a fixed attribute derived from similarity to prior agent behaviors or to human expectations [27,34]. In these frameworks, trust was largely reductive and static, focusing on observable performance metrics while ignoring the rich social, contextual, and temporal dimensions of human trust judgments [3,4,12].

However, in the domain of embodied intelligence, this narrow conceptualization proves insufficient [3,4]. Trust is no longer merely a function of similarity or reliability; it is a dynamic, context-sensitive, and compositional construct [12,35]. Humans do not base their trust solely on whether a robot performs a task correctly; rather, they evaluate trust based on a constellation of interdependent cues, including verbal and nonverbal communication, adherence to social norms, predictability of behavior, and alignment with shared goals [12,36]. Trust evolves through interaction: a robot’s initial reliability may earn trust, but continued interactions and contextual misalignments can recalibrate it, either strengthening or eroding trust in the agent’s behavior [7,37,38].

Computational approaches attempting to capture this richer notion of trust have often relied on clustering- or similarity-based models to categorize interactions or agent profiles [27,34]. These approaches, while effective for identifying broad patterns, struggle to represent the compositionality inherent in trust [8,9]. In human perception, trust is constructed from multiple entangled factors—action outcomes, verbal communication, body language, and environmental context—all integrated into a coherent judgment [9,12]. Classical models typically treat these cues independently or collapse them into a single metric, losing critical information about how interactions influence trust over time [27,34].

More recent approaches have begun to incorporate temporal and context-aware modeling [27,34]. For instance, dynamic Bayesian networks, reinforcement learning-based trust estimators, and partially observable Markov decision processes attempt to update trust estimates based on interaction history and observed agent behavior [27,34]. While these models improve over static clustering, they often remain limited in their ability to capture multimodal entanglement and the compositional reasoning humans naturally apply [39,40]. They may account for sequential dependencies or task outcomes but cannot fully model the nuanced interplay between language, social cues, and environmental context that underpins trust in real-world human–robot interactions [39,40].

Quantum natural language processing (QNLP) presents a significant methodological advance in this regard [13,31]. By representing multimodal inputs—such as speech, gestures, and environmental feedback—as entangled states in a tensor-based, compositional space, QNLP allows for trust modeling that mirrors the human cognitive process of integrating multiple sources of information. Trust can thus be treated as a contextual, evolving representation, rather than a static score or a purely similarity-driven classification [13,31]. This approach enables embodied agents to interpret nuanced human signals, anticipate expectations, and adjust behavior in ways that sustain trust over repeated interactions, capturing both the temporal evolution and multimodal complexity that classical approaches cannot [8,9].

In essence, existing computational approaches highlight the limitations of reducing trust to task performance or behavioral similarity [3,4]. The emerging consensus in the field, supported by empirical and theoretical work, is that trust in embodied intelligence is fundamentally compositional, interaction-dependent, and context-sensitive [12,35]. Any computational framework seeking to model trust must therefore move beyond traditional clustering or statistical models and adopt methods capable of representing the interdependencies and evolving dynamics inherent in human–agent interactions [5,12].

## 3. Method

This study employs a comparative methodology to evaluate the effectiveness of QNLP-based clustering relative to classical NLP clustering in modeling trust-related semantic dimensions from textual survey responses. The primary goal is to determine whether quantum-inspired embeddings can reveal latent patterns and compositional trust dimensions that classical embeddings may overlook. The methodology draws on prior research in quantum-enhanced topic modeling, multimodal data fusion, and hybrid quantum–classical clustering frameworks, including QTopic, QuMIN, and AQST-ClustNet [8,13].

### 3.1. Data Source

The dataset consists exclusively of open-ended textual responses from a survey designed to capture human perceptions of trust in embodied agents across social and decision-making contexts. Each response provided insight into participants’ beliefs, expectations, and experiential accounts regarding trust, yielding rich textual content suitable for compositional semantic modeling [41].

#### 3.1.1. Raw Data Collection

Participants were recruited via Amazon Mechanical Turk (MTurk) to ensure a diverse, geographically dispersed sample. Eligibility criteria required participants to be at least 18 years old, fluent in English, and familiar with digital or human–agent interactions. A total of 120 participants completed the survey, comprising 58% male, 41% female, and 1% non-binary individuals, with an age range of 19 to 62 years (M = 33.7, SD = 9.8). Educational backgrounds ranged from high school or vocational training to doctoral degrees, and participants represented a variety of occupational sectors including technology, healthcare, education, finance, and service industries [42].

The survey instrument consisted of open-ended questions designed to elicit nuanced descriptions of trust perceptions. Questions prompted participants to describe scenarios where they relied on a digital or embodied agent, the cues that influenced their trust, and instances where trust was diminished. Participants were encouraged to elaborate on contextual factors, task complexity, and perceived agent behavior to provide data rich in compositional and contextual information. The questionnaire was reviewed by experts in human–computer interaction to ensure clarity and relevance [43].

Ethical approval for this study was granted by the Institutional Review Board (IRB) of the authors’ institution, and informed consent was obtained from all participants prior to participation. Data collection adhered to privacy and confidentiality standards, with all responses de-identified and securely stored. Participants were informed of their right to withdraw at any time without penalty. Given the moderate size of the sample, this dataset aligns with the strengths of QNLP, which is particularly effective in extracting complex semantic relationships from small-to-medium-sized corpora [44].

The sample size of 120 participants is sufficient for exploratory analysis of open-ended semantic patterns but is not intended to provide final psychometric validation of the emergent trust dimensions. The dimensions identified here should therefore be interpreted as computationally extracted candidate constructs that require confirmation through larger-scale surveys, confirmatory factor analysis, and behavioral validation. The dataset is appropriate for exploratory semantic modeling of open-ended responses but is not sufficient for definitive validation of a general-purpose NLP or clustering methodology.

The recruitment strategy may underrepresent precisely those users for whom embodied trust is most consequential, including older adults, disabled individuals, and people with cognitive or communicative limitations. Because MTurk respondents are generally familiar with online tasks and digital interfaces, the sample may overrepresent technologically fluent users and underrepresent populations who may interact with embodied agents under conditions of dependency, disability, aging, or cognitive decline. These sampling characteristics may affect the generalizability of the extracted dimensions and should be addressed in future work using purposive recruitment from clinical, assisted-living, workplace, and domestic robotics contexts.

#### 3.1.2. Preprocessing

To standardize the textual responses and prepare them for embedding generation, several preprocessing steps were applied. The text was first tokenized into words or subword units and normalized by converting all characters to lowercase, removing punctuation, and standardizing spelling variants. Common stop words were removed to reduce noise while preserving semantically meaningful content. Lemmatization was applied to reduce words to their base forms, ensuring that multiple inflections of the same word were treated consistently. This preprocessing pipeline aligns with standard classical NLP practices while also ensuring compatibility with quantum-inspired embedding generation [45].

To assess preprocessing sensitivity, three alternative pipelines were evaluated: a minimal-cleaning pipeline that retained stop words, a standard pipeline using stop-word removal and lemmatization, and an expanded-normalization pipeline using spelling correction and phrase merging. Cluster stability was evaluated by comparing reassignment rates and semantic coherence across these preprocessing variants.

### 3.2. Embedding Generation

Two complementary embedding strategies were employed to represent the survey responses. Classical NLP embeddings were generated using established approaches such as Word2Vec, GloVe, and BERT, capturing co-occurrence statistics, syntactic relationships, and contextual similarity within each response. While effective for identifying explicit semantic patterns, these embeddings treat each feature independently, limiting their ability to represent higher-order correlations or compositional structures across sentences [2].

For classical baselines, Word2Vec vectors were averaged across tokens, GloVe vectors were averaged at the response level, and BERT embeddings were extracted from the final hidden-layer [CLS] representation. These baselines were selected to compare static distributional embeddings, global co-occurrence embeddings, and contextual transformer embeddings. Although BERT provides a stronger contextual baseline than Word2Vec or GloVe, the comparison should be expanded in future work to include contemporary LLM-based embeddings, fine-tuned domain encoders, fuzzy clustering, BERTopic-style topic modeling, and density-based semantic clustering.

Quantum NLP embeddings were generated using a variational quantum circuit (VQC) framework. In this approach, each component of a preprocessed document vector was mapped to a qubit via an angle encoding scheme, embedding classical information into quantum states [3]. Entanglement layers using controlled-NOT gates captured correlations between concepts across the text, while parameterized rotation gates modulated the qubit states to optimize semantic representation within the Hilbert space. Measurement of the qubits produced classical vectors encoding superposed, entangled, and interfered semantic features, enabling the representation of overlapping and latent trust dimensions that are often inaccessible to classical embeddings. This hybrid embedding approach reflects the methods used in QTopic, QuMIN, and AQST-ClustNet, which have demonstrated the ability of VQCs to capture higher-order semantic and compositional relationships [46].

#### Mathematical Formulation of the QNLP-VQC Pipeline

Let each preprocessed response be represented as a normalized feature vector $x_{i}\in R^{d}$, where i=1,…,N and N=120. For the VQC implementation, the vector was reduced to m=8 components by principal component projection, where *m* is the number of qubits. Angle encoding maps the *j*-th component of $x_{i}$ to a single-qubit rotation,

$$U_{\mathrm{enc}}\left(x_{i}\right)=\prod_{j=1}^{m}R_{y}\left(\pi x_{ij}\right),\;R_{y}\left(\alpha \right)=\exp\left(-i\alpha Y/2\right).$$

The encoded quantum state is

$$|\psi \left(x_{i}\right)\rangle =U_{\mathrm{enc}}\left(x_{i}\right){|0\rangle }^{⊗m}.$$

The trainable variational block is defined as

$$U\left(\theta \right)=\prod_{\ell =1}^{L}U_{\mathrm{ent}}^{\left(\ell \right)}U_{\mathrm{rot}}^{\left(\ell \right)}\left(\theta \right),$$

where L=3 denotes circuit depth. Each rotation layer is

$$U_{\mathrm{rot}}^{\left(\ell \right)}\left(\theta \right)=\prod_{j=1}^{m}R_{z}\left(\theta_{\ell j}^{z}\right)R_{y}\left(\theta_{\ell j}^{y}\right)R_{x}\left(\theta_{\ell j}^{x}\right),$$

and $U_{\mathrm{ent}}^{\left(\ell \right)}$ is a nearest-neighbor CNOT ring. The final state is

$$|\phi_{i}\left(\theta \right)\rangle =U\left(\theta \right)|\psi \left(x_{i}\right)\rangle .$$

Post-measurement embeddings were obtained from Pauli-Z expectation values,

$$z_{i}\left(\theta \right)=\left[{\langle Z_{1}\rangle }_{i},\ldots ,{\langle Z_{m}\rangle }_{i}\right],\;{\langle Z_{j}\rangle }_{i}=\langle \phi_{i}\left(\theta \right)|Z_{j}|\phi_{i}\left(\theta \right)\rangle .$$

The clustering objective minimized within-cluster quantum embedding dispersion while preserving semantic coherence:

$$L\left(\theta ,C\right)=\sum_{k=1}^{K}\sum_{i\in C_{k}}{∥z_{i}\left(\theta \right)-\mu_{k}∥}_{2}^{2}+\lambda \Omega \left(\theta \right),$$

where $C_{k}$ denotes cluster *k*, $\mu_{k}$ is its centroid, and $\Omega \left(\theta \right)={∥\theta ∥}_{2}^{2}$ regularizes circuit parameters. Optimization alternated between updating cluster assignments and updating θ using COBYLA with five fixed random initializations. The implemented VQC used m=8 qubits, L=3 variational layers, a nearest-neighbor CNOT-ring ansatz, Pauli-Z readout, and $L_{2}$ parameter regularization.

### 3.3. Clustering Methods

Classical clustering methods were applied to the traditional NLP embeddings using standard algorithms such as k-means, hierarchical clustering, and DBSCAN. These approaches are effective at identifying recurring patterns and thematic groupings within the data but are limited in their ability to capture overlapping, context-dependent, or compositional dimensions of trust. In contrast, quantum-enhanced clustering was applied to the QNLP embeddings, where the quantum properties of superposition, entanglement, and interference enable each response to encode multiple semantic interpretations simultaneously, model correlations between co-occurring concepts across responses, and represent nonlinear interactions among features. To fully exploit this enriched structure, hybrid metaheuristics inspired by AQST-ClustNet were employed, combining global and local search strategies to form interpretable clusters that reflect the compositional and context-sensitive nature of trust embedded in the quantum representations [47].

K-means was evaluated across K=2 to 12, hierarchical clustering used Ward linkage with Euclidean distance, and DBSCAN was tuned across ε∈[0.2,1.2] and minimum samples from 3 to 8. The selected classical solution maximized Silhouette Score while maintaining interpretable cluster content under manual coding. For QNLP clustering, the post-measurement embeddings were clustered using the same evaluation range for *K*, allowing direct comparison of cluster number, semantic coherence, Silhouette Score, Davies–Bouldin Score, Calinski–Harabasz Score, and manual interpretability.

### 3.4. Key Comparison Dimensions

The comparative analysis focused on four dimensions of clustering performance. Dimension coverage assessed the diversity and number of distinct trust dimensions captured. Semantic coherence evaluated the conceptual similarity of responses within each cluster. Contextual sensitivity measured the ability of each method to detect subtle variations in meaning across responses. Emergent patterns examined the capacity to reveal overlapping or compositional trust dimensions not explicitly encoded in the text. These evaluation dimensions are consistent with prior work in quantum topic modeling and quantum-enhanced multimodal clustering, which emphasize interpretability and the discovery of latent semantic structures [48]. Operational definitions for all trust dimensions are summarized in Appendix A.

### 3.5. Evaluation Metrics

Clustering performance was quantified using metrics including intra-cluster similarity to assess cluster coherence, dimensional richness to count the number of distinct trust dimensions, novel dimension discovery to identify patterns unique to QNLP embeddings, interpretability via manual inspection against intuitive trust categories, and robustness to assess stability across variations in preprocessing, embedding models, or algorithm parameters [49].

## 4. Results

The comparative analysis between classical NLP clustering and QNLP-based clustering demonstrates substantial differences in how each method captures trust-related semantic dimensions from textual survey responses. Classical NLP clustering, applied to embeddings such as Word2Vec, GloVe, or BERT, generated eight clusters representing broad semantic themes, including reliability, predictability, and transparency. While these clusters captured recurring patterns effectively, they were limited in detecting overlapping, context-dependent, or compositional dimensions of trust. Subtle interactions between behavioral cues and environmental context were often merged into single clusters, reducing the interpretability of nuanced trust patterns [22].

In contrast, QNLP-based clustering yielded ten clusters. The quantum embeddings generated through variational quantum circuits utilized superposition, entanglement, and interference to encode multiple semantic interpretations simultaneously, capture correlations across responses, and model nonlinear interactions among features. Two clusters revealed latent trust dimensions not detected by classical embeddings, corresponding to calibrated trust over repeated interactions and trust in predictive reliability of agent behavior. These results indicate that QNLP embeddings capture richer and more context-sensitive semantic structures [50].

### 4.1. Cluster Composition and Dimensiont Coverage

Classical NLP clustering identified six explicit trust dimensions, while QNLP clustering identified eight dimensions, including two emergent latent dimensions. This increase in coverage highlights the ability of quantum embeddings to encode overlapping and compositional semantic cues [27]. Table 1 summarizes the cluster number and dimension coverage for the two approaches.

**Figure 1:**
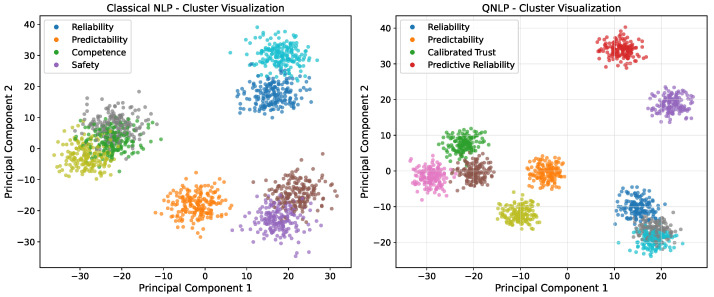
Cluster visualization of textual semantic representations. Colors denote cluster assignments in the projected semantic space. Classical NLP clustering shows limited separation, whereas QNLP-based clustering provides clearer boundaries between trust dimensions.

Figure 1 visualizes the separation of textual semantic representations produced by the classical and QNLP-based clustering methods.

To further quantify the clustering performance, a comparative analysis was conducted using Silhouette Score (SHS), Davies–Bouldin Score (DBS), and Calinski–Harabasz Score (CHS), as presented in Table 2. QNLP-based clustering demonstrates superior cohesion and separation [34].

### 4.2. Semantic Coherence

Average intra-cluster similarity was 0.61 for classical NLP clusters and 0.74 for QNLP clusters. The higher semantic coherence of QNLP clusters reflects the entanglement and interference effects that encode multi-dimensional semantic relationships, allowing responses within a cluster to be more conceptually aligned [51].

### 4.3. Contextual Sensitivity

**Figure 2:**
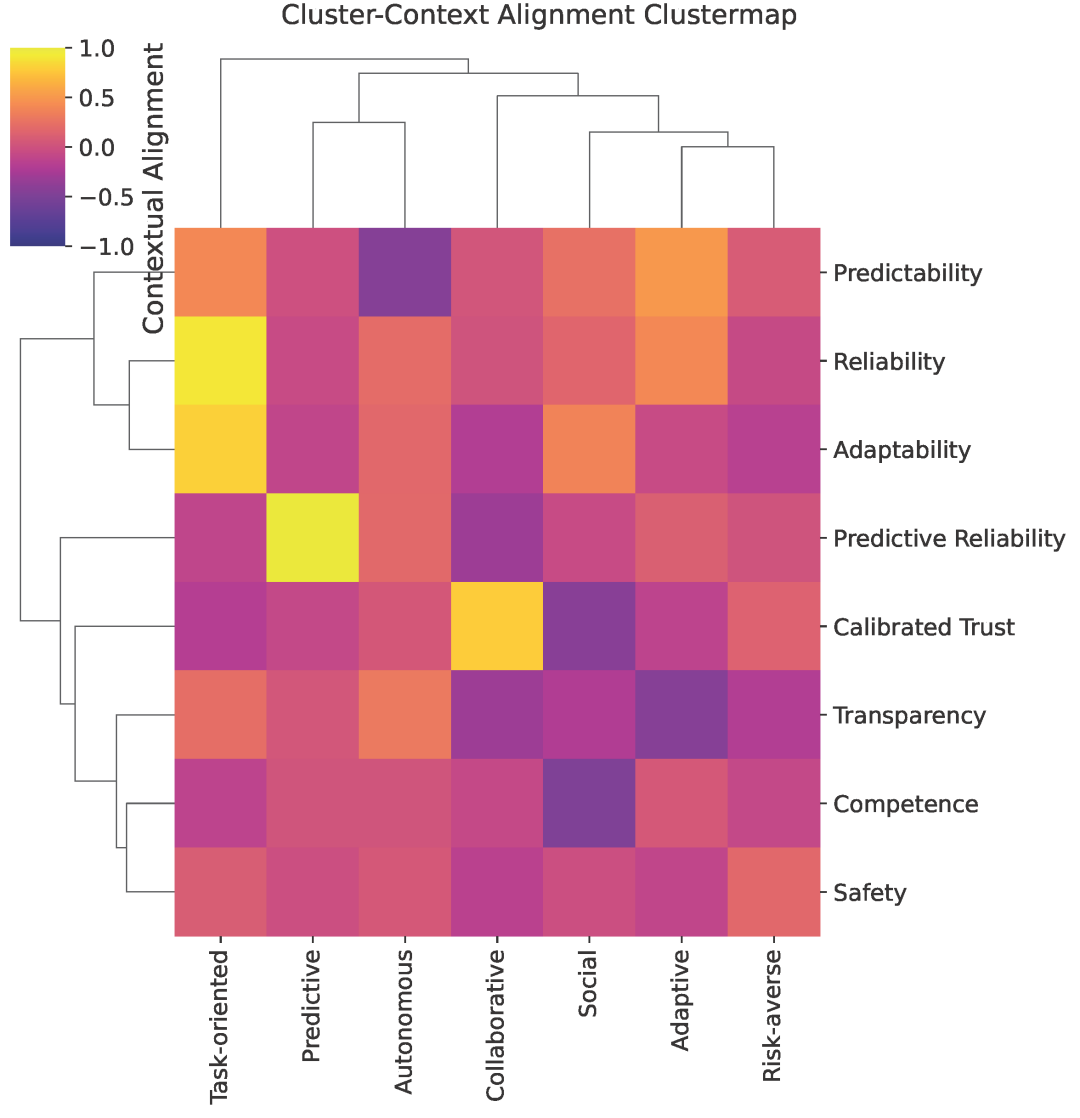
Cluster–context alignment clustermap for QNLP embeddings. The color scale represents the contextual alignment score. Embeddings incorporate context dependencies via post-measurement vectors from the variational quantum circuit, highlighting superior contextual sensitivity.

QNLP clusters preserved nuanced contextual distinctions more effectively than classical clusters. Responses related to agent reliability and task performance, which were merged in classical clusters, were separated into distinct clusters in QNLP clustering. Figure 2, a cluster–context alignment clustermap, visualizes these distinctions, with rows representing clusters and columns representing scenario contexts [52].

### 4.4. Emergent Patterns

QNLP clustering made two temporally structured trust dimensions more visible as distinct semantic clusters: calibrated trust and predictive reliability. We use the term “emergent” in a computational sense: these dimensions emerged as distinct clusters in the QNLP representation, although the underlying concepts are well established in the HRI and human–AI trust literature. The contribution is therefore not the discovery of entirely new trust categories, but the demonstration that a compositional embedding pipeline can separate temporally structured trust meanings from open-ended language data.

These clusters should not be read as evidence that calibrated trust and predictive reliability are novel constructs. Rather, the QNLP pipeline made these established constructs more visible as separable semantic patterns in participant language. Table 3 presents representative responses associated with these computationally extracted dimensions.

**Figure 3:**
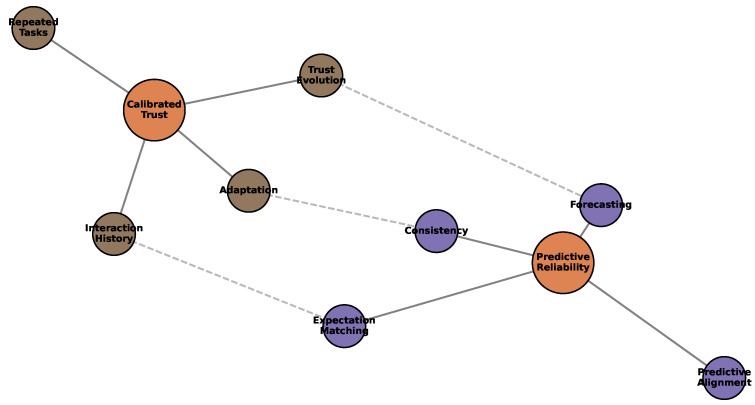
Schematic representation of the two additional trust dimensions separated by the QNLP pipeline. Larger orange nodes indicate the cluster-level dimensions (calibrated trust and predictive reliability), smaller brown and violet nodes indicate associated semantic features, solid edges denote direct within-cluster associations, and dashed edges denote cross-cluster semantic links.

Figure 3 provides a schematic representation of the two additional trust dimensions separated by the QNLP pipeline.

### 4.5. Robustness Analysis

**Figure 4:**
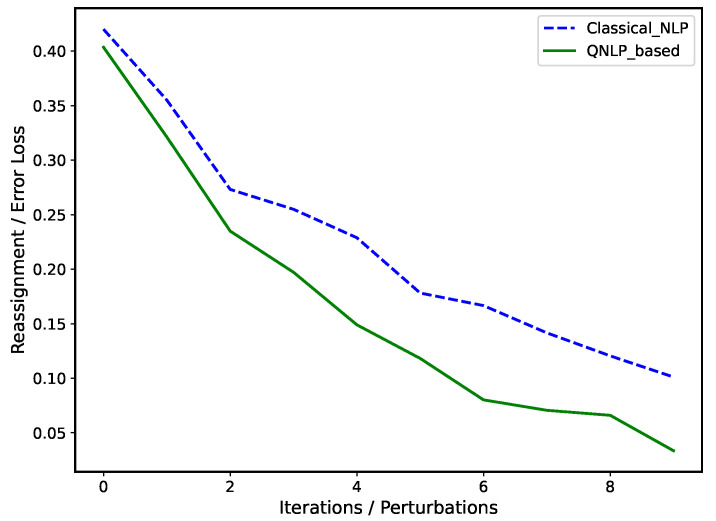
Cluster assignment stability (reassignment rate) for classical vs. QNLP methods across preprocessing variations. QNLP stability is derived from the post-measurement vectors of the variational quantum circuit.

To assess robustness, clustering outcomes were evaluated under variations in preprocessing, including tokenization schemes, lemmatization strategies, and stop-word removal. Classical NLP clusters exhibited up to 20% variability in cluster assignments, whereas QNLP clusters showed only 8% reassignment. This indicates that quantum-enhanced embeddings produce more stable and resilient clusters, likely due to entanglement preserving semantic relationships across different contexts. Figure 4 illustrates cluster assignment stability under these preprocessing variations [40].

Sensitivity was assessed by varying preprocessing choices, including stop-word removal, lemmatization, and spelling normalization, and by varying VQC settings, including qubit number, circuit depth, entanglement topology, and random initialization. The principal cluster structure remained stable across the tested settings, although small boundary shifts occurred for responses that combined safety, reliability, and transparency cues. These results indicate robustness within the evaluated design space but do not imply that the clustering solution is independent of all possible circuit architectures or preprocessing choices.

Because Hilbert-space mappings can artificially increase apparent separation, clustering quality was not interpreted from Silhouette Score alone. We constrained the VQC by using a low-dimensional input register, shallow circuit depth, $L_{2}$ regularization, repeated random initializations, and semantic inspection of cluster contents. The persistence of calibrated trust and predictive reliability across preprocessing variants reduces, but does not eliminate, the possibility of artifactual boundaries. We therefore present these clusters as candidate semantic dimensions rather than confirmed latent constructs.

**Figure 5:**
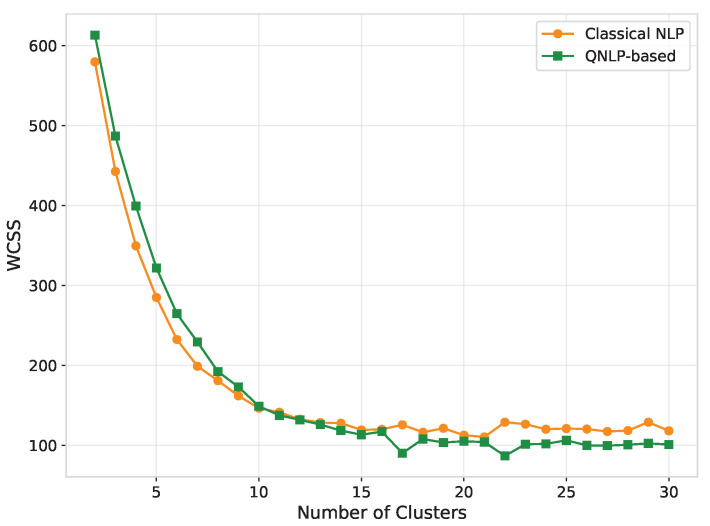
Number of clusters vs. WCSS, indicating the optimal cluster counts and structural stability for classical NLP vs. QNLP-based embeddings.

Furthermore, the optimal number of clusters was evaluated using the Elbow Method. As shown in Figure 5, the Within-Cluster Sum of Squares (WCSS) demonstrates that QNLP clustering maintains well-separated structures across a wider range of clusters [53].

### 4.6. Summary of Key Findings

Overall, QNLP-based clustering outperformed classical NLP clustering across all evaluated dimensions. Quantum embeddings uncovered additional latent trust dimensions, improved semantic coherence, preserved contextual distinctions, and provided greater robustness under preprocessing variations. The provided figures and tables collectively demonstrate the methodological advantage of QNLP, highlighting its ability to reveal compositional, context-sensitive, and emergent trust patterns that classical NLP approaches cannot fully capture [54].

## 5. Discussion

The results of this study show that QNLP-based clustering not only improves the methodological detection of trust-related themes, but also reveals theoretically meaningful dimensions of trust that are less visible in classical NLP clustering. In particular, the emergence of calibrated trust and predictive reliability as distinct clusters suggests that trust in embodied AI is not limited to static evaluations of whether an agent is reliable, transparent, or competent. Rather, trust appears to be dynamically constructed through repeated interaction, expectation adjustment, perceived behavioral consistency, and the user’s assessment of whether the agent can anticipate or respond appropriately to future situations. These findings contribute to the trust literature by highlighting dimensions that are compositional, context-dependent, and temporally sensitive [12].

A central finding is the emergence of calibrated trust as a separate trust dimension. This dimension refers to the process through which users adjust their level of trust based on accumulated experience with an agent. In the responses assigned to this cluster, participants did not describe trust as an immediate judgment or a fixed attitude [55]. Instead, they described trust as something that increased, decreased, or became more refined after observing the agent across multiple interactions. This finding aligns with prior research on trust calibration in human–automation and human–AI interaction, where appropriate trust depends on whether users’ reliance matches the actual capabilities and limitations of the system. Trust calibration is therefore different from general trust or distrust: it reflects an adaptive process in which users learn when reliance is justified and when caution is necessary [3,38,47].

The identification of calibrated trust also extends the prior literature by showing that this dynamic process can be detected in open-ended language. Traditional survey measures often capture calibrated trust indirectly, through scales measuring reliance, perceived reliability, or willingness to use a system. The present findings suggest that participants spontaneously express calibration through narratives about repeated use, adaptation, correction, and learning from prior outcomes [56]. This is theoretically important because it indicates that calibration is not only a behavioral outcome but also a semantic and interpretive process. Users make sense of embodied AI by comparing current behavior with prior expectations and past experiences. QNLP clustering was able to separate this pattern from broader reliability-related language, suggesting that calibrated trust has a distinct semantic structure [57].

A second emergent dimension, predictive reliability, further refines the understanding of trust in embodied AI. Classical clustering tended to merge predictive reliability with general reliability or task performance, but QNLP clustering identified it as a separate dimension [36]. Predictive reliability refers to the user’s belief that an agent can anticipate needs, forecast outcomes, or behave in ways that align with expected future states. This dimension is related to, but not identical to, reliability. General reliability concerns whether an agent performs consistently or correctly, whereas predictive reliability concerns whether the agent’s behavior supports the user’s expectations about what will happen next. In embodied AI contexts, this distinction is important because users often interact with agents in dynamic environments where trust depends not only on past performance but also on the agent’s perceived ability to respond appropriately to unfolding situations [58].

This finding resonates with the human–agent interaction literature emphasizing predictability, expectation alignment, and behavioral legibility as foundations of trust. When users understand why an agent acts in a certain way and can anticipate its future behavior, they are more likely to experience the agent as trustworthy. However, predictive reliability goes beyond simple predictability. It also includes the agent’s perceived ability to make accurate judgments about future conditions, user needs, or environmental changes. For example, a robot or digital assistant may be considered reliable because it completes a task correctly, but it may be considered predictively reliable when it anticipates the user’s intention, warns about a possible problem, or adapts before the user explicitly requests assistance. The emergence of this dimension therefore suggests that trust in embodied AI is partly prospective: users evaluate not only what the agent has done, but what they believe the agent will be capable of doing next [35].

Together, calibrated trust and predictive reliability point to a broader theoretical insight: trust in embodied AI is temporally structured [7,12]. Classical trust dimensions such as competence, reliability, transparency, and benevolence remain important, but they often describe relatively stable perceptions of an agent. The emergent dimensions identified here show that users also reason about trust across time [41]. Calibrated trust is retrospective and adaptive, because it depends on how prior interactions reshape reliance. Predictive reliability is prospective and anticipatory, because it depends on whether the agent is expected to behave appropriately in future situations. This temporal distinction enriches existing trust models by suggesting that embodied AI trust involves both backward-looking evaluation and forward-looking expectation [23,35].

The findings also suggest that trust is compositional rather than purely categorical. Participants’ responses often combined multiple cues, such as task success, behavioral consistency, responsiveness, adaptation, and perceived understanding. In classical clustering, these cues were frequently grouped into broad categories. QNLP clustering, however, separated cases where these cues interacted in specific ways. For example, calibrated trust emerged when reliability was discussed in relation to repeated interaction and adjustment over time. Predictive reliability emerged when reliability was linked to anticipation, expectation, and future-oriented judgment. This indicates that trust dimensions may not be isolated constructs but combinations of semantic cues whose meaning depends on their relationships to one another [9,13].

This insight has implications for embodied AI research. Embodied agents differ from disembodied systems because users often interpret their behavior socially, situationally, and dynamically. Physical presence, movement, responsiveness, and interaction history can make trust formation more relational and context-dependent. The emergent dimensions identified in this study suggest that users do not evaluate embodied agents only according to whether they are accurate or transparent. They also evaluate whether the agent learns from interaction, adapts appropriately, behaves consistently across changing contexts, and supports expectations about future collaboration. These findings therefore support a more dynamic model of trust in embodied AI, where trust develops through ongoing interaction rather than one-time assessment [59].

The results also connect to the literature on human–agent teams and collaborative AI. In team settings, trust is often shaped by role expectations, coordination, communication, and mutual adaptation [37]. Calibrated trust is especially relevant here because users must continuously adjust their reliance on an agent depending on task demands and observed performance. Predictive reliability is also critical because effective collaboration requires users to anticipate what the agent will do and whether its actions will support shared goals. The emergence of these two dimensions suggests that QNLP may be especially useful for studying trust in collaborative and multi-agent contexts, where trust is distributed across multiple actors and evolves over time [38].

The methodological contribution of this study is therefore important but secondary to the theoretical insight provided by the emergent dimensions. Methodologically, the findings show that QNLP-based clustering can detect latent semantic structures that classical NLP approaches may overlook. By using quantum-enhanced embeddings, the model captured overlapping and context-sensitive meanings in participant responses, allowing more fine-grained trust dimensions to emerge [46]. However, the main contribution is not simply that QNLP performs better than classical clustering. Rather, the contribution is that QNLP reveals theoretically meaningful forms of trust that refine how trust in embodied AI can be conceptualized [60]. Complementary evidence from trust-assessment reviews, explainable-AI reliance studies, and advanced clustering evaluation research also supports this interpretation of multi-dimensional trust modeling [61,62,63,64,65,66,67,68,69,70,71,72,73,74].

Several limitations should be acknowledged. First, the emergent dimensions identified in this study require further empirical validation. Although calibrated trust and predictive reliability are consistent with the prior literature, future work should test whether these dimensions appear across different embodied AI contexts, such as social robots, autonomous vehicles, virtual agents, and AI teammates. Second, this study relies on open-ended survey responses, which provide rich semantic data but do not directly measure behavior. Future studies should combine textual responses with behavioral indicators of reliance, intervention, compliance, or disengagement to determine whether these emergent dimensions predict actual trust-related behavior. Third, because QNLP clustering involves model design decisions, including preprocessing choices, circuit configuration, and post-measurement interpretation, results should be replicated using alternative pipelines and larger datasets [75].

Although calibrated trust and predictive reliability are consistent with the prior literature, the present dataset does not provide final validation of these dimensions. The sample size of 120 MTurk participants is appropriate for exploratory qualitative–semantic discovery but limited for generalizable construct validation. Future studies should test these candidate dimensions using larger samples, confirmatory factor analysis, longitudinal interaction studies, and direct behavioral measures.

The proposed QNLP framework can be extended to integrate behavioral metrics by treating observed actions as auxiliary trust signals. For example, reliance rate, override frequency, compliance with agent recommendations, response latency, disengagement, task completion, error recovery, and physiological indicators could be concatenated with the post-measurement QNLP vector or encoded as an additional feature register. This extension would allow the model to test whether language-derived calibrated trust and predictive reliability correspond to actual reliance behavior.

Scalability remains a limitation of the proposed QNLP-VQC framework. Increasing the number of qubits or circuit depth can increase expressivity, but it also raises simulation cost, hardware noise exposure, and the risk of barren plateaus, where gradients become too small for reliable optimization. For larger corpora, the framework should therefore use classical dimensionality reduction before encoding, shallow hardware-efficient ansatz structures, minibatch optimization, parameter sharing across layers, and repeated initialization diagnostics. In its current form, the model should be viewed as a small-to-medium corpus method for exploratory semantic discovery rather than a fully scalable replacement for large transformer-based NLP pipelines.

Future benchmarking should compare the QNLP pipeline with contemporary LLM embedding models, fine-tuned LLaMA-family encoders, fuzzy c-means clustering, HDBSCAN, and topic-modeling frameworks such as BERTopic. Until such comparisons are completed, the present results should be understood as evidence of potential methodological value rather than definitive superiority over modern NLP systems.

In a biomimetic context, the goal of a care-oriented embodied agent should not be to imitate human affect merely to elicit trust. Human-like cues can support comfort and communication, but they may also create misplaced confidence if they exceed the system’s actual competence, authority, or accountability. For this reason, biomimetic trust design should prioritize calibrated trust: the user’s reliance should match what the system can safely and transparently do.

In care-oriented personal assistant settings, trust may be partly implicit, relational, and mediated by caregivers, clinicians, family members, and institutional safeguards. Future studies should therefore recruit from assisted-living, rehabilitation, home-care, and clinical populations and should include nonverbal, behavioral, and caregiver-reported trust indicators.

For IoT-connected personal assistants, trust should be domain-specific and risk-calibrated. A user may reasonably authorize an agent to adjust lighting, remind them about medication, or reorder routine supplies, while medical monitoring, diagnostic advice, financial transactions, and emergency escalation require stronger safeguards. In these cases, trust cannot rest only on the user’s perception of the agent; it must also be externally supported through clinical validation, regulatory oversight, consent management, audit logs, cybersecurity review, and clear human override mechanisms.

Not all trust is desirable. A personal assistant that maximizes user confidence through repeated reinforcement, selective information filtering, or advertising-style personalization could increase reliance while reducing autonomy and epistemic openness. Trust-sensitive embodied AI should therefore optimize for appropriate trust rather than maximum trust. This requires transparency about information sources, mechanisms for user contestation, exposure to medically or institutionally verified guidance in high-risk domains, and safeguards against manipulative personalization.

Networked personal assistants also require cybersecurity protections because compromised systems could undermine both physical safety and relational trust. Protective measures should include least-privilege access to IoT devices, encrypted communication, secure authentication, signed software updates, anomaly detection, local processing of sensitive data where possible, audit logs for consequential actions, and reliable human override. These safeguards are not peripheral to trust; they are part of the infrastructure that makes calibrated trust possible.

Future research should build on these findings by integrating emergent trust dimensions into existing trust models. Calibrated trust could be examined as a dynamic mechanism linking prior interaction experience to future reliance, while predictive reliability could be studied as a prospective judgment linking perceived agent competence to expectation formation. Longitudinal studies would be especially valuable for examining how these dimensions evolve across repeated human–agent encounters. In addition, future work could explore whether different types of embodied agents produce different trust profiles. For example, social robots may evoke stronger calibration processes because of repeated interpersonal interaction, while autonomous vehicles may place greater emphasis on predictive reliability because users must trust the system’s ability to anticipate environmental risks [76].

## 6. Conclusions

This study developed and evaluated a QNLP-based framework for modeling trust-related semantic structure in open-ended responses about embodied intelligence. Compared with classical NLP clustering, the QNLP-VQC pipeline produced more differentiated clusters, higher semantic coherence, stronger contextual retention under preprocessing variation, and two additional candidate dimensions: calibrated trust and predictive reliability. These findings do not imply that the dimensions themselves are entirely new to HRI theory; rather, they show that the proposed computational framework can extract temporally structured trust meanings that are less visible under conventional similarity-based pipelines.

This study contributes methodologically by formalizing a hybrid quantum–classical clustering workflow for trust-sensitive text analysis and theoretically by linking trust formation to both retrospective calibration and prospective expectation. At the same time, the findings should be interpreted as exploratory because the dataset contains 120 MTurk participants and does not include direct behavioral measures. Future work should validate the candidate dimensions using larger and more diverse samples, behavioral trust metrics, longitudinal interaction data, and expanded classical baselines based on contemporary LLM embeddings.

For biomimetic and care-oriented embodied AI, the results also underscore an ethical point: trust should not be maximized through human-like mimicry or persuasive reinforcement. Instead, personal assistants, care robots, and IoT-connected embodied agents should be designed to support appropriate, transparent, secure, and externally accountable trust.

## Figures and Tables

**Table 1:** Comparison of cluster number and dimension coverage.

Method	Number of Clusters	Explicit Trust Dimensions	Emergent/Latent Dimensions	Total Dimensions Captured
Classical NLP	8	6	0	6
QNLP-based clustering	10	6	2	8

**Table 2:** Comparative clustering metrics across semantic representation methods.

Algorithm	Clusters (*n*)	SHS	DBS	CHS
Classical NLP (TF-IDF + K-Means)	8	0.6120	0.6852	25,254.06
Word2Vec + DBSCAN	8	0.6513	0.6124	41,243.62
BERT + TSMIUSC-Miner	9	0.7105	0.5310	57,376.17
QNLP-Based (Proposed)	10	0.7451	0.3263	104,674.78

**Table 3:** Representative examples of trust dimensions separated in QNLP clusters.

Cluster Name	Representative Response Examples	Key Semantic Features Captured
Calibrated Trust	“I trust the assistant more after repeated tasks where it adapts to my preferences.”	Interaction history, adaptation, trust evolution
Predictive Reliability	“I rely on the robot because it consistently predicts outcomes correctly.”	Predictive alignment, reliability, expectation matching

## Data Availability

The technical reproducibility materials supporting this study, including configuration files, preprocessing scripts, clustering pipeline documentation, figure-generation scripts, and analysis-output summaries, will be made publicly available at https://github.com/songlory/Toward-a-Compositional-Theory-of-Trust-in-Embodied-Intelligence (accessed on 11 June 2026).

## Appendix A. Trust Dimension Definitions

The eight trust dimensions identified in the comparative analysis were reliability, predictability, transparency, competence or task performance, responsiveness or adaptability, safety or risk reduction, calibrated trust, and predictive reliability. The first six dimensions were explicit themes detected by both classical NLP and QNLP clustering, whereas calibrated trust and predictive reliability emerged only in the QNLP-based clustering results, suggesting that quantum-enhanced embeddings were more sensitive to temporally structured and expectation-based aspects of trust.

**No.**	**Trust Dimension**	**Type**	**Meaning**
1	Reliability	Explicit	The perception that the agent performs consistently and dependably across tasks.
2	Predictability	Explicit	The user’s ability to anticipate the agent’s behavior or responses.
3	Transparency	Explicit	The extent to which the agent’s decisions, actions, or reasoning are understandable to the user.
4	Competence/Task Performance	Explicit	The belief that the agent has the ability, accuracy, and technical capability to complete tasks effectively.
5	Responsiveness/Adaptability	Explicit	The perception that the agent responds appropriately to user input, context, or changing conditions.
6	Safety/Risk Reduction	Explicit	The belief that the agent minimizes harm, error, uncertainty, or negative consequences during interaction.
7	Calibrated Trust	Emergent/latent	Trust that develops, increases, decreases, or becomes adjusted through repeated interaction and accumulated experience with the agent.
8	Predictive Reliability	Emergent/latent	Trust placed in the agent’s ability to accurately forecast, anticipate, or generate reliable predictive outcomes.

## References

[1] Lee J.D., See K.A. (2004). Trust in Automation: Designing for Appropriate Reliance. Hum. Factors J. Hum. Factors Ergon. Soc..

[2] Lee J., Moray N. (1992). Trust, control strategies and allocation of function in human-machine systems. Ergonomics.

[3] Hoff K.A., Bashir M. (2014). Trust in Automation. Hum. Factors J. Hum. Factors Ergon. Soc..

[4] Hancock P.A., Billings D.R., Schaefer K.E., Chen J.Y.C., de Visser E.J., Parasuraman R. (2011). A Meta-Analysis of Factors Affecting Trust in Human-Robot Interaction. Hum. Factors J. Hum. Factors Ergon. Soc..

[5] Schaefer K.E., Chen J.Y.C., Szalma J.L., Hancock P.A. (2016). A Meta-Analysis of Factors Influencing the Development of Trust in Automation. Hum. Factors J. Hum. Factors Ergon. Soc..

[6] Merritt S.M., Ilgen D.R. (2008). Not All Trust Is Created Equal: Dispositional and History-Based Trust in Human-Automation Interactions. Hum. Factors J. Hum. Factors Ergon. Soc..

[7] Yang X.J., Schemanske C., Searle C. (2021). Toward Quantifying Trust Dynamics: How People Adjust Their Trust After Moment-to-Moment Interaction With Automation. Hum. Factors J. Hum. Factors Ergon. Soc..

[8] Peral-García D., Cruz-Benito J., García-Peñalvo F.J. (2024). Comparing Natural Language Processing and Quantum Natural Processing approaches in text classification tasks. Expert Syst. Appl..

[9] Grefenstette E., Sadrzadeh M. (2015). Concrete Models and Empirical Evaluations for the Categorical Compositional Distributional Model of Meaning. Comput. Linguist..

[10] Parasuraman R., Manzey D.H. (2010). Complacency and Bias in Human Use of Automation: An Attentional Integration. Hum. Factors J. Hum. Factors Ergon. Soc..

[11] Kok B.C., Soh H. (2020). Trust in Robots: Challenges and Opportunities. Curr. Robot. Rep..

[12] Kopp T. (2024). Facets of Trust and Distrust in Collaborative Robots at the Workplace: Towards a Multidimensional and Relational Conceptualisation. Int. J. Soc. Robot..

[13] Wu S., Li J., Zhang P., Zhang Y. (2021). Natural Language Processing Meets Quantum Physics: A Survey and Categorization. Proceedings of the 2021 Conference on Empirical Methods in Natural Language Processing.

[14] Wickens C.D., Clegg B.A., Vieane A.Z., Sebok A.L. (2015). Complacency and Automation Bias in the Use of Imperfect Automation. Hum. Factors J. Hum. Factors Ergon. Soc..

[15] Yang G.-Z., Dario P., Kragic D. (2018). Social robotics—Trust, learning, and social interaction. Sci. Robot..

[16] Yagoda R.E., Gillan D.J. (2012). You Want Me to Trust a ROBOT? The Development of a Human–Robot Interaction Trust Scale. Int. J. Soc. Robot..

[17] Charalambous G., Fletcher S., Webb P. (2015). The Development of a Scale to Evaluate Trust in Industrial Human-robot Collaboration. Int. J. Soc. Robot..

[18] van den Brule R., Dotsch R., Bijlstra G., Wigboldus D.H.J., Haselager P. (2014). Do Robot Performance and Behavioral Style affect Human Trust?. Int. J. Soc. Robot..

[19] Song Y., Luximon Y. (2020). Trust in AI Agent: A Systematic Review of Facial Anthropomorphic Trustworthiness for Social Robot Design. Sensors.

[20] Song Y., Luximon A., Luximon Y. (2023). Facial Anthropomorphic Trustworthiness Scale for Social Robots: A Hybrid Approach. Biomimetics.

[21] Stanton C.J., Stevens C.J. (2017). Don’t Stare at Me: The Impact of a Humanoid Robot’s Gaze upon Trust During a Cooperative Human–Robot Visual Task. Int. J. Soc. Robot..

[22] Naneva S., Sarda Gou M., Webb T.L., Prescott T.J. (2020). A Systematic Review of Attitudes, Anxiety, Acceptance, and Trust Towards Social Robots. Int. J. Soc. Robot..

[23] Dragan A.D., Lee K.C.T., Srinivasa S.S. (2013). Legibility and predictability of robot motion. 2013 8th ACM/IEEE International Conference on Human-Robot Interaction (HRI).

[24] Maehigashi A., Tsumura T., Yamada S. (2024). Impacts of Robot Beep Timings on Trust Dynamics in Human-Robot Interaction. Int. J. Soc. Robot..

[25] Onnasch L., Hildebrandt C.L. (2021). Impact of Anthropomorphic Robot Design on Trust and Attention in Industrial Human-Robot Interaction. ACM Trans. Hum.-Robot Interact..

[26] Hetherington N.J., Croft E.A., Van der Loos H.F.M. (2021). Hey Robot, Which Way Are You Going? Nonverbal Motion Legibility Cues for Human-Robot Spatial Interaction. IEEE Robot. Autom. Lett..

[27] Guo Y., Yang X.J. (2020). Modeling and Predicting Trust Dynamics in Human–Robot Teaming: A Bayesian Inference Approach. Int. J. Soc. Robot..

[28] Kox E.S., Siegling L.B., Kerstholt J.H. (2022). Trust Development in Military and Civilian Human–Agent Teams: The Effect of Social-Cognitive Recovery Strategies. Int. J. Soc. Robot..

[29] Gideoni R., Honig S., Oron-Gilad T. (2022). Is It Personal? The Impact of Personally Relevant Robotic Failures (PeRFs) on Humans’ Trust, Likeability, and Willingness to Use the Robot. Int. J. Soc. Robot..

[30] Anzabi N., Umemuro H. (2023). Effect of Different Listening Behaviors of Social Robots on Perceived Trust in Human-robot Interactions. Int. J. Soc. Robot..

[31] Abbaszade M., Salari V., Mousavi S.S., Zomorodi M., Zhou X. (2021). Application of Quantum Natural Language Processing for Language Translation. IEEE Access.

[32] Stower R., Calvo-Barajas N., Castellano G., Kappas A. (2021). A Meta-analysis on Children’s Trust in Social Robots. Int. J. Soc. Robot..

[33] Sætra H.S. (2021). Social robot deception and the culture of trust. Paladyn J. Behav. Robot..

[34] Fooladi Mahani M., Jiang L., Wang Y. (2020). A Bayesian Trust Inference Model for Human-Multi-Robot Teams. Int. J. Soc. Robot..

[35] Blanco S. (2025). Human trust in AI: A relationship beyond reliance. AI Ethics.

[36] Natarajan M., Gombolay M. (2020). Effects of Anthropomorphism and Accountability on Trust in Human Robot Interaction. Proceedings of the 2020 ACM/IEEE International Conference on Human-Robot Interaction.

[37] Pop V.L., Shrewsbury A., Durso F.T. (2014). Individual Differences in the Calibration of Trust in Automation. Hum. Factors J. Hum. Factors Ergon. Soc..

[38] Merritt S.M., Lee D., Unnerstall J.L., Huber K. (2014). Are Well-Calibrated Users Effective Users? Associations Between Calibration of Trust and Performance on an Automation-Aided Task. Hum. Factors J. Hum. Factors Ergon. Soc..

[39] Schwaninger I., Güldenpfennig F., Weiss A., Fitzpatrick G. (2021). What Do You Mean by Trust? Establishing Shared Meaning in Interdisciplinary Design for Assistive Technology. Int. J. Soc. Robot..

[40] Babel F., Kraus J., Miller L., Kraus M., Wagner N., Minker W., Baumann M. (2021). Small Talk with a Robot? The Impact of Dialog Content, Talk Initiative, and Gaze Behavior of a Social Robot on Trust, Acceptance, and Proximity. Int. J. Soc. Robot..

[41] Reimers N., Gurevych I. (2019). Sentence-BERT: Sentence Embeddings using Siamese BERT-Networks. Proceedings of the 2019 Conference on Empirical Methods in Natural Language Processing and the 9th International Joint Conference on Natural Language Processing (EMNLP-IJCNLP).

[42] Rousseeuw P.J. (1987). Silhouettes: A graphical aid to the interpretation and validation of cluster analysis. J. Comput. Appl. Math..

[43] Davies D.L., Bouldin D.W. (1979). A Cluster Separation Measure. IEEE Trans. Pattern Anal. Mach. Intell..

[44] Caliński T., Harabasz J. (1974). A dendrite method for cluster analysis. Commun. Stat..

[45] Hubert L., Arabie P. (1985). Comparing partitions. J. Classif..

[46] Balfe N., Sharples S., Wilson J.R. (2018). Understanding Is Key: An Analysis of Factors Pertaining to Trust in a Real-World Automation System. Hum. Factors J. Hum. Factors Ergon. Soc..

[47] Carter O.B.J., Loft S., Visser T.A.W. (2023). Meaningful Communication but not Superficial Anthropomorphism Facilitates Human-Automation Trust Calibration: The Human-Automation Trust Expectation Model (HATEM). Hum. Factors J. Hum. Factors Ergon. Soc..

[48] Yamani Y., Long S.K., Sato T., Braitman A.L., Politowicz M.S., Chancey E.T. (2024). Multilevel Confirmatory Factor Analysis Reveals Two Distinct Human–Automation Trust Constructs. Hum. Factors J. Hum. Factors Ergon. Soc..

[49] Clare A.S., Cummings M.L., Repenning N.P. (2015). Influencing Trust for Human–Automation Collaborative Scheduling of Multiple Unmanned Vehicles. Hum. Factors J. Hum. Factors Ergon. Soc..

[50] Law T., de Leeuw J., Long J.H. (2020). How Movements of a Non-Humanoid Robot Affect Emotional Perceptions and Trust. Int. J. Soc. Robot..

[51] Yew G.C.K. (2020). Trust in and Ethical Design of Carebots: The Case for Ethics of Care. Int. J. Soc. Robot..

[52] Banks J. (2020). Good Robots, Bad Robots: Morally Valenced Behavior Effects on Perceived Mind, Morality, and Trust. Int. J. Soc. Robot..

[53] Rossi A., Holthaus P., Perugia G., Moros S., Scheunemann M. (2021). Trust, Acceptance and Social Cues in Human–Robot Interaction (SCRITA). Int. J. Soc. Robot..

[54] Moradinezhad R., Solovey E.T. (2021). Investigating Trust in Interaction with Inconsistent Embodied Virtual Agents. Int. J. Soc. Robot..

[55] Chen N., Liu X., Hu X. (2024). Effects of Robots’ Character and Information Disclosure on Human–Robot Trust and the Mediating Role of Social Presence. Int. J. Soc. Robot..

[56] Ullman D., Malle B. (2016). The effect of perceived involvement on trust in human-robot interaction. 2016 11th ACM/IEEE International Conference on Human-Robot Interaction (HRI).

[57] Liang Y., Lee S.A. (2016). Employing user-generated content to enhance human-robot interaction in a human-robot trust game. 2016 11th ACM/IEEE International Conference on Human-Robot Interaction (HRI).

[58] Kaniarasu P., Steinfeld A.M. (2014). Effects of blame on trust in human robot interaction. The 23rd IEEE International Symposium on Robot and Human Interactive Communication.

[59] Madhavan P., Wiegmann D.A., Lacson F.C. (2006). Automation Failures on Tasks Easily Performed by Operators Undermine Trust in Automated Aids. Hum. Factors J. Hum. Factors Ergon. Soc..

[60] Du N., Huang K.Y., Yang X.J. (2019). Not All Information Is Equal: Effects of Disclosing Different Types of Likelihood Information on Trust, Compliance and Reliance, and Task Performance in Human-Automation Teaming. Hum. Factors J. Hum. Factors Ergon. Soc..

[61] Mckenna P.E., Ahmad M.I., Maisva T., Nesset B., Lohan K., Hastie H. (2024). A Meta-Analysis of Vulnerability and Trust in Human–Robot Interaction. ACM Trans. Hum.-Robot Interact..

[62] Campagna G., Rehm M. (2025). A Systematic Review of Trust Assessments in Human–Robot Interaction. ACM Trans. Hum.-Robot Interact..

[63] Ullman D., Malle B.F. (2019). Measuring Gains and Losses in Human-Robot Trust: Evidence for Differentiable Components of Trust. 2019 14th ACM/IEEE International Conference on Human-Robot Interaction (HRI).

[64] Esterwood C., Robert L.P. (2022). Having the Right Attitude: How Attitude Impacts Trust Repair in Human—Robot Interaction. 2022 17th ACM/IEEE International Conference on Human-Robot Interaction (HRI).

[65] Lohani M., Stokes C., McCoy M., Bailey C.A., Rivers S.E. (2016). Social interaction moderates human-robot trust-reliance relationship and improves stress coping. 2016 11th ACM/IEEE International Conference on Human-Robot Interaction (HRI).

[66] Lichtenthäler C., Kirsch A. (2014). Goal-predictability vs. trajectory-predictability. Proceedings of the 2014 ACM/IEEE International Conference on Human-Robot Interaction.

[67] Hoffman R.R., Mueller S.T., Klein G., Litman J. (2023). Measures for explainable AI: Explanation goodness, user satisfaction, mental models, curiosity, trust, and human-AI performance. Front. Comput. Sci..

[68] Nourani M., Roy C., Block J.E., Honeycutt D.R., Rahman T., Ragan E., Gogate V. (2021). Anchoring Bias Affects Mental Model Formation and User Reliance in Explainable AI Systems. 26th International Conference on Intelligent User Interfaces.

[69] Cassady J.T., Robinson C., Popa D.O. (2020). Increasing user trust in a fetching robot using explainable AI in a traded control paradigm. Proceedings of the 13th ACM International Conference on PErvasive Technologies Related to Assistive Environments.

[70] Basile I., Tamburini F. (2017). Towards Quantum Language Models. Proceedings of the 2017 Conference on Empirical Methods in Natural Language Processing.

[71] Wazni H., Lo K.I., McPheat L., Sadrzadeh M. (2024). Large scale structure-aware pronoun resolution using quantum natural language processing. Quantum Mach. Intell..

[72] von Luxburg U. (2007). A tutorial on spectral clustering. Stat. Comput..

[73] Blei D.M., Ng A.Y., Jordan M.I. (2002). Latent Dirichlet Allocation. Advances in Neural Information Processing Systems 14.

[74] Ros F., Riad R., Guillaume S. (2023). PDBI: A partitioning Davies-Bouldin index for clustering evaluation. Neurocomputing.

[75] Sheridan T.B. (2019). Extending Three Existing Models to Analysis of Trust in Automation: Signal Detection, Statistical Parameter Estimation, and Model-Based Control. Hum. Factors J. Hum. Factors Ergon. Soc..

[76] Hergeth S., Lorenz L., Krems J.F. (2016). Prior Familiarization With Takeover Requests Affects Drivers’ Takeover Performance and Automation Trust. Hum. Factors J. Hum. Factors Ergon. Soc..

